# Efficient bit labeling in factorization machines with annealing for traveling salesman problem

**DOI:** 10.1038/s41598-025-10064-4

**Published:** 2025-07-24

**Authors:** Shota Koshikawa, Aruto Hosaka, Tsuyoshi Yoshida

**Affiliations:** Information Technology R&D Center, Mitsubishi Electric Corporation, Kanagawa, 247-8501 Japan

**Keywords:** Annealing, Black box optimization, Factorization machines, Traveling salesman problem, Quantum physics, Quantum simulation

## Abstract

To efficiently determine an optimum parameter combination in a large-scale problem, it is essential to convert the parameters into available variables in actual machines. Specifically, quadratic unconstrained binary optimization problems are solved using machine learning, for example, factorization machines with annealing, which convert a raw parameter to binary variables. This study investigates the dependence of the convergence speed and accuracy on the binary labeling method, which can influence the cost function shape and thus the probability of being captured at a local minimum solution. By exemplifying the traveling salesman problem (TSP), we propose and evaluate Gray labeling, which correlates the Hamming distance in binary labels with the traveling distance. Through numerical simulation of the TSP at a limited number of iterations, the Gray labeling shows fewer local minima percentages and shorter traveling distances compared with natural labeling.

## Introduction

Combinatorial optimization problems have attracted significant attention across various domains, including logistics, transportation systems, and manufacturing^1, 2^, owing to their wide range applications and potentials for cost reduction and efficiency improvement. The computational complexity of these problems is generally classified to NP hardness, making it challenging to approach an optimal solution with a reasonable number of computational resources^3, 4^. Renowned for its computational complexity as an NP-hard problem, the traveling salesman problem (TSP) serves as a cornerstone in numerous fields, and being vigorously researched^5, 6^.

The complexity of these difficult problems can be reduced by combining machine learning. In particular, factorization machines with annealing (FMA)^7–12^ is a useful technique for black-box optimization^13–17^. The FMA employs factorization machines (FM)^18^ with binary variables as a surrogate model. Because the model takes the form of a quadratic unconstrained binary optimization (QUBO), Ising machines can be utilized to efficiently obtain a good solution for the model^19^.

The performance of a QUBO solver depends on the labeling method, that is, how the actual non-binary variables are replaced by binary variables available in the solver. Although the labeling method is the key to characterizing how frequently a solver is captured in local solutions, research on it is limited^20–22^. It aims to create a smoother energy landscape by assigning bit states with short Hamming distances to binary variable configurations that are close to the solution space. By ensuring that similar solutions are represented by bit states with short Hamming distances, we can achieve more efficient optimization.

According to the situation described above, this study contributes to QUBO formulation of TSP with a reduced number of bits by employing FMA, the proposal of Gray labeling useful for avoiding local solutions based on the idea of *similar bits for similar routes*, the proposal of a metric for local solution characterization, and a comparison of conventional natural labeling and Gray labeling.

## Preliminaries

This section reviews the fundamentals of FMA and TSP.

### Factorization machines with annealing

Rendle proposed an FM model for a high prediction performance with efficient high-order feature interactions. This prediction is given by the sum of the linear and quadratic-order interaction terms^18^

1 $$\begin{aligned} \begin{aligned} y = w_0 +\sum _{\textsf{i}=1}^{\textsf{n}}{w_{\textsf{i}}x_{\textsf{i}}} + \sum _{\textsf{i}=1}^{\textsf{n}}\sum _{\textsf{j}=\textsf{i}+1}^{\textsf{n}} \langle \textbf{v}_{\textsf{i}}, \textbf{v}_{\textsf{j}}\rangle x_{\textsf{i}} x_{\textsf{j}} . \end{aligned} \end{aligned}$$

The input data are represented as feature vector $\textbf{x} = (x_1, x_2, \ldots , x_\textsf{n})$ of $\textsf{n}$ real-valued features, and *y* is an objective variable. $w_0$ is the global bias, $w_\textsf{i}$ is the weight of the $\textsf{i}$-th feature, and weight vector $\textbf{w} = (w_1, \cdots , w_\textsf{n})$. $\textbf{v}_\textsf{i}$ is the $\textsf{k}$-dimensional latent vector of the $\textsf{i}$-th feature and the vector sequence $\textbf{V} = (\textbf{v}_1, \cdots , \textbf{v}_\textsf{n})$. The interaction between features $x_\textsf{i}$ and $x_\textsf{j}$ is approximated by inner product $\langle \textbf{v}_\textsf{i}, \textbf{v}_\textsf{j}\rangle$. The model parameters $(w_0, \textbf{w}, \textbf{V})$ are optimized to minimize the error between the predicted and actual values of the training data. In this study, we set the dimension of the latent vector $\textsf{k}$ to 12, used Adam for optimizing the FM model estimation, with a learning rate of $1.0 \times 10^{-2}$ and 1000 epochs.

Unlike support vector machines, FM uses factorized parameters to model all variable interactions. In conventional polynomial models, it is necessary to prepare the individual interaction parameters for each combination, such as $w_{\textsf{i}\textsf{j}}x_{\textsf{i}}x_{\textsf{j}}$. However, $x_{\textsf{i}} x_{\textsf{j}}$ becomes zero for sparse data, making it almost impossible to calculate $w_{\textsf{i}\textsf{j}}$. By contrast, FM represents the magnitude of the interaction of $x_{\textsf{i}}x_{\textsf{j}}$ as $\langle \textbf{v}_\textsf{i}, \textbf{v}_\textsf{j}\rangle$; that is, it is no longer mutually independent of each $w_{\textsf{i}\textsf{j}}$. Therefore, even if one or both interaction components are zero, if there is a non-zero component of $x_\textsf{i}$ or $x_\textsf{j}$ somewhere, parameters $\textbf{v}_\textsf{i}$ and $\textbf{v}_\textsf{j}$ can be learned. This implies that the FM can indirectly learn interaction effects, even from data without target interaction components. Thus, the FM is robust in handling sparse data and has a relatively low computational cost^18^. This renders it useful for high-dimensional sparse data applications.

**Fig. 1:**
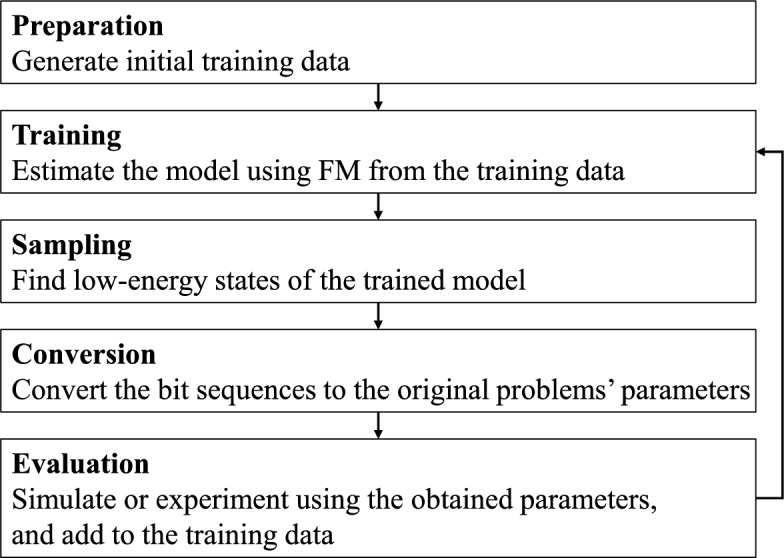
The process of FMA.

FM can be combined with an optimization method of annealing^7–12^, where the combination is called FMA. The model equation of FM with binary variables can be rewritten in the QUBO form

2 $$\begin{aligned} y = w_0 + \sum _{\textsf{i}=1}^{\textsf{n}}\sum _{\textsf{j}=\textsf{i}}^{\textsf{n}} Q_{\textsf{i}\textsf{j}}x_{\textsf{i}}x_{\textsf{j}} , \end{aligned}$$

where $Q = (Q_{\textsf{i}\textsf{j}})$ is an $\textsf{n} \times \textsf{n}$ QUBO matrix, $Q_{\textsf{i}\textsf{i}}= w_\textsf{i}$, $Q_{\textsf{i}\textsf{j}}= \langle \textbf{v}_\textsf{i}, \textbf{v}_\textsf{j}\rangle$. We now explain the optimization method for black-box optimization problems using the FMA. The FMA comprises four main phases repeated in an iterative cycle^7^.

- *Training* The FM model is trained using the available training data. A solution candidate of the single bit sequence $\textbf{b}$ were randomly generated, and the pairs of $\textbf{b}$ and corresponding energy (objective variables) were added for the initial training. The parameters of the FM are optimized to minimize the mean-squared error between the predicted values and the actual energy values.
- *Sampling* New bit sequences are generated from the trained FM model, focusing on samples with low predicted energy values. Because the FM model is formulated as a QUBO, quantum or classical annealing techniques can be employed to find low-energy states, which corsrespond to good samples.
- *Conversion* The bit sequences generated in the sampling are converted back to the original optimization problem’s parameters. This aspect is detailed in the bit labeling methods’ section.
- *Evaluation* The costs are simulated or experimented using parameters obtained at the previous iteration, and the pairs of the binarized parameters and the corresponding energy are used to update the training data.

The FMA approach iterates through these four phases multiple times, gradually refining the approximation of the black box function, in this case QUBO, and improving the quality of the solutions as shown in Fig. 1. After a given number of iterations, the best sample obtained during the optimization process is returned as the final solution.

### Traveling salesman problem

TSP is one of the most widely studied combinatorial optimization problems^23, 24^, which attempts to find the shortest route that visits all predefined points exactly once and returns to the origin. This can be extended to various optimization problems, such as component assembly sequences in manufacturing, delivery routes in logistics, and data transmission paths in telecommunication networks.

Regarding the complexity of the TSP, as the number of cities, *N*, increases, the total number of possible routes increases exponentially and reaches $(N-1)!$, e.g., $1.3 \times 10^{12}$ routes for $N=16$ . It is impractical to perform a brute-force search in the case of a large *N*. Various algorithms have been proposed to determine the optimal solution for the TSP, including the well-known dynamic programming and branch-and-bound algorithms, which reach the exact solution^25, 26^. Specifically , the Held-Karp algorithm^25^ has a time complexity of $O(N^2 2^N)$. However, these algorithms are difficult to apply to a case with a large *N*; thus, they are often combined with an approximation method such as the greedy algorithm^27^, local search method^28^, genetic algorithm^29^, ant colony optimization^30^, and quantum/simulated annealing^31^.

In this study, $N=5$–16 cities were placed in rectangular coordinates $(\alpha , \beta )$, where $\alpha$ and $\beta$ ($\in [0,1]$) were randomly obtained. Each city has unique integer index $i \in \{ 0, 1, \ldots , N-1 \}$. The departure and destination cities were indexed as 0. An arbitrary route is described as $\textbf{r} = (r_1, r_2, \cdots , r_{N-1})$ except for the 0-th city. The objective is to minimize the distance as follows:

3 $$\begin{aligned} \begin{aligned} d(\textbf{r}) = \sum _{j=0}^{N-1}{\sqrt{(\alpha _{r_{j+1}} - \alpha _{r_{j}})^2 + (\beta _{r_{j+1}} - \beta _{r_{j}})^2}}, \end{aligned} \end{aligned}$$

where $r_0=r_N=0$ according to the definition.

## Bit labeling methods

This study treats the TSP with FMA; therefore, any variables in the TSP must be redescribed by binary variables only. This section explains the labeling methods for converting TSP route $\textbf{r}$ into single- bit sequence $\textbf{b}$. In a well-known labeling method, $N^2$ bits are employed to formulate *N*-city TSP, resulting in a quadratic Hamiltonian^19^. Recent studies using improved labeling reduced the number of bits to $N \textrm{log}{N}$^32, 33^. In this study, $\textrm{log}$ denotes the logarithm of base 2.

**Table 1:** Examples of natural and Gray labeling.

Route	Natural				Gray			
$\textbf{r}$	$m(=\underline{m})$	$\underline{m}(\ne m)$	$\textbf{b} (= \underline{\textbf{b}})$	$\underline{\textbf{b}} (\ne \textbf{b})$	$|\mathcal {S}| (= |\underline{\mathcal {S}}|)$	$|\underline{\mathcal {S}}| (\ne |\mathcal {S}|)$	$\textbf{b} (= \underline{\textbf{b}})$	$\underline{\textbf{b}} (\ne \textbf{b})$
(1,2,3,4)	0	24	00000	11000	(0,0,0)	(0,3,0)	00000	01000
(1,2,4,3)	1	25	00001	11001	(0,0,1)	(0,3,1)	00001	01001
(1,3,2,4)	2	26	00010	11010	(0,1,0)	–	00100	–
(1,3,4,2)	3	27	00011	11011	(0,1,1)	–	00101	–
(1,4,2,3)	4	28	00100	11100	(0,0,2)	(0,3,2)	00011	01011
(1,4,3,2)	5	29	00101	11101	(0,1,2)	–	00111	–
(2,1,3,4)	6	30	00110	11110	(1,0,0)	(1,3,0)	10000	11000
(2,1,4,3)	7	31	00111	11111	(1,0,1)	(1,3,1)	10001	11001
(2,3,1,4)	8	–	01000	–	(1,1,0)	–	10100	–
(2,3,4,1)	9	–	01001	–	(1,1,1)	–	10101	–
(2,4,1,3)	10	–	01010	–	(1,0,2)	(1,3,2)	10011	11011
(2,4,3,1)	11	–	01011	–	(1,1,2)	–	10111	–
(3,1,2,4)	12	–	01100	–	(0,2,0)	–	01100	–
(3,1,4,2)	13	–	01101	–	(0,2,1)	–	01101	–
(3,2,1,4)	14	–	01110	–	(1,2,0)	–	11100	–
(3,2,4,1)	15	–	01111	–	(1,2,1)	–	11101	–
(3,4,1,2)	16	–	10000	–	(0,2,2)	–	01111	–
(3,4,2,1)	17	–	10001	–	(1,2,2)	–	11111	–
(4,1,2,3)	18	–	10010	–	(0,0,3)	(0,3,3)	00010	01010
(4,1,3,2)	19	–	10011	–	(0,1,3)	–	00110	–
(4,2,1,3)	20	–	10100	–	(1,0,3)	(1,3,3)	10010	11010
(4,2,3,1)	21	–	10101	–	(1,1,3)	–	10110	–
(4,3,1,2)	22	–	10110	–	(0,2,3)	–	01110	–
(4,3,2,1)	23	–	10111	–	(1,2,3)	–	11110	–

The forward labeling performs $\textbf{r}\rightarrow \textbf{b}$, and the inverse one does $\mathbf {\underline{b}}\rightarrow \textbf{r}$. Each set of $\mathbf {\underline{m}}$, $\mathbf {\underline{b}}$, and $|\mathcal {\underline{S}}|$ is a superset of *m*, $\textbf{b}$, and $|\mathcal {S}|$, respectively. Only the extended eight elements are shown in the 3-rd, 5-th, 7-th, and 9-th columns.

**Table 2:** Examples of Gray labeling: (a) route $\textbf{r} =$ (7, 5, 3, 6, 8, 1, 4, 2) and (b) route $\textbf{r} =$ (5, 7, 3, 6, 8, 1, 4, 2).

(a) Route $\textbf{r} =$ (**7, 5**, 3, 6, 8, 1, 4, 2)				(b) Route $\textbf{r} =$ (**5, 7**, 3, 6, 8, 1, 4, 2)			
*i*	$\mathcal {S}_i$	$|\mathcal {S}_i|$	$g_i(|\mathcal {S}_i|)$	*i*	$\mathcal {S}_i$	$|\mathcal {S}_i|$	$g_i(|\mathcal {S}_i|)$
1	(Always $\varnothing$)	(Always 0)	(Always 0)	1	(Always $\varnothing$)	(Always 0)	(Always 0)
2	$\varnothing$	0	0	2	$\varnothing$	0	0
3	{1,2}	2	11	3	{1,2}	2	11
4	{2}	1	01	4	{2}	1	01
**5**	**{1,2,3,4}**	**4**	**110**	**5**	**{1,2,3,4}**	**4**	**110**
6	{1,2,4}	3	010	6	{1,2,4}	3	010
**7**	**{1,2,3,4,5,6}**	**6**	**101**	**7**	**{1,2,3,4,6}**	**5**	**111**
8	{1,2,4}	3	010	8	{1,2,4}	3	010
$l_G(\textbf{r})=$ 01101**110**010**101**010				$l_G(\textbf{r})=$ 01101**110**010**111**010			

Significant values are in bold.

### Bit labelings in channel coding

Bit labelings are essential in channel coding for spectrally efficient and reliable communication. Although the logical layer treats bits, the channel requires symbols, where the bits to symbols mapping rule is provided to minimize bit errors caused by symbol errors as less as possible. It is preferable to provide similar labels with small Hamming distances to neighboring symbols with small Euclidean distances. A well-known method is binary (reflected) Gray coding^34^, where $2^{\textsf{m}}$-ary pulse amplitudes are labeled with $\textsf{m}$ bits, such that every Hamming distance between the nearest amplitudes is exactly 1. For example, amplitudes $\{3, 1, -1, -3\}$ are labeled as $\{00, 01, 10, 11 \}$ with natural coding and $\{00, 01, 11, 10 \}$ with Gray coding. This study extends the established concept of Gray coding to our binary labeling method, which could be the key to avoiding local solutions in optimization problems.

### Forward labeling

Let $l_\textrm{N}(\cdot )$ and $l_\textrm{G}(\cdot )$ denote the bit labeling functions obtained by applying natural and Gray labelings, respectively. The output obtained by inputting route $\textbf{r}$ provides bit sequence $\textbf{b}$. Table 1 shows an example of $N=5$, which includes forward labeling $\textbf{r}\rightarrow \textbf{b}$ and inverse labeling $\mathbf {\underline{b}}\rightarrow \textbf{r}$. Based on this definition, the bit sequence set is generally larger than the route set. Thus, we employ $\textbf{b}$ for a bit sequence with a one-to-one correspondence to $\textbf{r}$ (used in forward labeling) and $\mathbf {\underline{b}}$ for an arbitrary combination of bits (used in inverse labeling).

Natural labeling directly corresponds $(N-1)!$ permutation cases in *N*-city TSP routes $\textbf{r}$ to nonnegative integers $m \in \{ 0, 1, \ldots , (N-1)!-1 \}$, where *m* is further described by a single-bit sequence $\textbf{b}$ of length $\ell _{\textrm{N}}=\lceil \log (N-1)! \rceil (=\lceil \sum _{i=2}^{N-1}{\textrm{log}i}\rceil )$, following the straight binary manner. $\textbf{b}$ is obtained by $\textbf{b}=n_{\ell _{\textrm{N}}}(m)$, where $n_{\cdot }(\cdot )$ is a function that obtains a bit sequence with length $\lambda$ from an arbitrary nonnegative integer $\gamma$, that is,

4 $$\begin{aligned} \begin{aligned} n_{\lambda }(\gamma ) = \sigma _{0 \le k < \lambda } (\eta _k(\gamma )). \end{aligned} \end{aligned}$$

$\eta _k(\gamma )$ is the function used to obtain the *k*-th bit from an arbitrary nonnegative integer $\gamma$ with a straight binary, that is,

5 $$\begin{aligned} \begin{aligned} \eta _{k}(\gamma ) = \textrm{mod} (\lfloor \gamma /2^k \rfloor , 2), \end{aligned} \end{aligned}$$

where $\textrm{mod}(\cdot ,\cdot )$ denotes the modulo function. $\sigma$ denotes the bit concatenation function from the most significant bit (the ($k_0-1$)-th bit) to the least significant bit (the 0-th bit); that is,

6 $$\begin{aligned} \begin{aligned} \sigma _{0 \le k < k_0}(b_k) = b_{k_0 -1} b_{k_1 -2} \ldots b_{1} b_{0} \end{aligned} \end{aligned}$$

with an arbitrary nonnegative integer $k_0$. The permutations are arranged in the lexicographical order. For example, as $N = 5$ and $\ell _{\textrm{N}}$ = 5, $l_\textrm{N}((1, 2, 3, 4)) = n_{5}(0) = 00000$, $l_\textrm{N}((1, 2, 4, 3)) = n_5(1) = 00001$, $l_\textrm{N}((1, 3, 2, 4)) = n_{5}(2) = 00010$, $\ldots$, $l_\textrm{N}((4,3,2,1)) = n_{5}(4!-1) = 10111$.

In contrast, our proposal for Gray labeling combines the inversion number and Gray coding. The inversion number is the concept of discrete mathematics and is related to the type of sort–the bubble sort–of sequences^35, 36^. Gray labeling consists mainly of the following two steps:

Step 1. For every *i*-th city ($i = 2, 3, \ldots , N-1$), identify the inversion cities. An inversion city is defined as any city with an index $j < i$ ($j = 1, 2, \ldots , N-2$) that is visited after the *i*-th city in the route (excluding the 0-th city). Form the set of these inversion cities, denoted as $\mathcal {S}_i$, and calcluate its size $|\mathcal {S}_i|$.

Step 2. Convert each $|\mathcal {S}_i|$ into a component bit sequence of length $\lceil \log i \rceil$ using Gray coding function $g_i(|\mathcal {S}_i|)$. Concatenate the component single-bit sequence with an order from $i=2$ to $N-1$ to a single-bit sequence with length $\ell _\textrm{G} = \sum _{i=2}^{N-1} \lceil \log i \rceil$.

This labeling method is explained using a small example; the city route $\textbf{r}=(7, 5, 3, 6, 8, 1, 4, 2)$ for $N=9$ shown in Table 2. Step 1 enumerates the inversion cities. For example, there are four smaller numbers (3, 1, 4, 2) after 5; thus, $\mathcal {S}_5=\{1, 2, 3, 4\}$ and $|\mathcal {S}_5|=4$, and no smaller numbers after 2; thus, $\mathcal {S}_2=\varnothing$ and $|\mathcal {S}_2|=0$, where $\varnothing$ denotes the empty set. By enumerating every inversion number from $i=2$ to $N-1$ in the same manner, $|\mathcal {S}| = (|\mathcal {S}_2|, |\mathcal {S}_3|, \ldots , |\mathcal {S}_8|) = (0, 2, 1, 4, 3, 6, 3)$ is obtained. Step 2 converts $|\mathcal {S}_i|$ into a component bit sequence using the Gray coding function

7 $$\begin{aligned} \begin{aligned} g_i(|\mathcal {S}_i|) = n_{\lambda }(|\mathcal {S}_i|) \oplus n_{\lambda }(\lfloor |\mathcal {S}_i| / 2 \rfloor ), \end{aligned} \end{aligned}$$

where $\lambda = \lceil \log i \rceil$. $\oplus$ denotes the bitwise exclusive OR operator. According to this definition, $g_2(|\mathcal {S}_2|) \rightarrow 0$, $g_3(|\mathcal {S}_3|) \rightarrow 11$, $\ldots$, $g_8(|\mathcal {S}_8|) \rightarrow 010$. These examples demonstrate the function outputs where each bit string is represented using the minimum length required to store the corresponding number of inversion cities for that specific city. Note that $i=1$ is ignored because 1 has no inversion numbers. Finally, every obtained sequence for *i* is concatenated from $i=2$ to $N-1=8$ into a single bit sequence $\textbf{b}=$ 01101110010101010 of length $\ell _\textrm{G} = \sum _{i=2}^{8} \lceil \log i \rceil = 17$. The conversion from $\textbf{r} \rightarrow \textbf{b}$ is injective but not surgective owing to redundant description of the binary variables.

### Inverse labeling

Let the inverse labeling function of $$l_\textrm{N}(\textbf{r}), l_\textrm{G}(\textbf{r})$$ be $$l_\textrm{N}^{-1}(\mathbf {\underline{b}}), l_\textrm{G}^{-1}(\mathbf {\underline{b}})$$, to a given single bit sequence. When we employ annealing machines to optimize binary variables, the obtained bit sequence $$\mathbf {\underline{b}}$$ can be arbitrary, resulting in $$2^{\ell }$$ possible cases with $$\ell$$ bits. Since $$2^{\ell }$$ generally exceeds the number of possible routes $$(N-1)!$$, we need a mechanism to map any arbitrary bit sequence $$\mathbf {\underline{b}}$$ to a valid route $$\textbf{r}$$. The forward labeling functions $$l_\textrm{N}$$ and $$l_\textrm{G}$$ (mapping $$\textbf{r} \rightarrow \textbf{b}$$) are both injective and surjective, allowing them to be inverted directly for valid bit sequences. However, to define the inverse mapping for arbitrary bit sequences $$\mathbf {\underline{b}} \rightarrow \textbf{r}$$, we must introduce an intermediate step. For natural labeling, we first convert $$\mathbf {\underline{b}}$$ to its integer representation $$\underline{m}=n_{\ell _{\textrm{N}}}^{-1}(\mathbf {\underline{b}})$$. We then compute $$m = \textrm{mod} (\underline{m}, (N-1)!)$$, ensuring the result is within the valid range. Finally, we can obtain a route $$\textbf{r}=l_{\textrm{N}}^{-1}(\textbf{b})$$, where $$\textbf{b}=n_{\ell _{\textrm{N}}}(m)$$. In Gray labeling, the inverse operation recovers route $$\textbf{r}=g_i^{-1}(|\underline{\mathcal {S}_i}|)$$, where $$|\underline{\mathcal {S}_i}| = \textrm{mod} (|\mathcal {S}_i|, i)$$. As an example in Table 1, for $$\mathbf {\underline{b}}=11011$$, the corresponding $$\underline{m}=27$$. In natural labeling, $$m=\textrm{mod}$$$$(27, (5-1)!)\,=\,3$$, and $$l_\textrm{N}^{-1}(11011)$$$$\,=\,l_\textrm{N}^{-1}(00011)$$$$\,=\,(1, 3, 4, 2)$$. In Gray labeling, $$|\mathcal {S}|$$$$\,=\,(|\mathcal {S}_2|,$$$$|\mathcal {S}_3|,$$$$|\mathcal {S}_4|)$$$$\,=\,(1, 3, 2)$$, and $$|\underline{\mathcal {S}}|$$$$\,=\,(|\underline{\mathcal {S}_2}|,$$$$|\underline{\mathcal {S}_3}|,$$$$|\underline{\mathcal {S}_4}|)$$$$\,=\,(1, 0, 2)$$. Therefore, $$l_\textrm{G}^{-1}(11011)$$$$\,=\,l_\textrm{G}^{-1}(10011)$$$$\,=\,(2, 4, 1, 3)$$.

The bit length for Gray labeling $\ell _\textrm{G}=\sum _{i=2}^{N-1}\lceil \log i \rceil$ is greater than or equal to that for natural labeling, $\ell _\textrm{N}= \lceil \log (N-1)! \rceil (=\lceil \sum _{i=2}^{N-1}{\textrm{log}i}\rceil )$. Lengths $\ell _\textrm{N}$ and $\ell _\textrm{G}$ are approximated as $O(\textrm{log}(N!)) = O(N\textrm{log}N)$. The proposed method for combining the inversion number and Gray coding originates from the idea of *similar bits for similar routes*. For a pair of similar routes, in the relationship of swapping two cities consecutively visited, the Hamming distance between their bit sequences is equal to 1 for the proposed Gray labeling. Let $r_j$ and $r_{j+1}$ be the indices for a pair of consecutive cities visited. In Gray labeling, $|\mathcal {S}_{r_{j+1}}|$ under $r_j < r_{j+1}$ is smaller by 1 than $|\mathcal {S}_{r_{j+1}}|$ under $r_j > r_{j+1}$ and the other $|\mathcal {S}_i|$ is maintained. When the resultant bit sequence pair is obtained from the difference in $|\mathcal {S}_{r_{j+1}}|$, the Hamming distance between them is guaranteed to be 1 with Gray coding and not guaranteed with natural coding. Table 2 shows an example of similar routes (a) $\textbf{r}=(7, 5, 3, 6, 8, 1, 4, 2)$ and (b) $\textbf{r}=(5, 7, 3, 6, 8, 1, 4, 2)$. In this case, only $|\mathcal {S}_7|$ is different from each other and the other $|\mathcal {S}_i|$ are identical, and the Hamming distance between their concatenated bit sequences is exactly 1.

## Local solution metric and analysis

The performance of an optimization solver is characterized by the balance of the solution quality and required computational resources, which can be translated into the Ising energy and the number of iterations for a solver based on an annealing machine. The proposed Gray labeling in the previous section is useful, specifically for avoiding local solutions. This section introduces the *local solution metric* for quantifying the expected performance without running the actual optimization procedure.

Our local solution metric is given by the number of local solutions normalized by the total number of solution candidates, as explained later. A solution is defined as a local solution if all nearest solutions (with a Hamming distance of one from the solution under examination) have worse or equal solution quality. Instead of $d(\textbf{r})$, let $d(\mathbf {\underline{b}})$ denote the total traveling distance in each route $\textbf{r} = l_{N}^{-1}(\mathbf {\underline{b}})$ for natural labeling or $\textbf{r} = l_{G}^{-1}(\mathbf {\underline{b}})$ for Gray labeling according to Eq. (3). The local solution flag is defined as

8 $$\begin{aligned} \begin{aligned} f(\mathbf {\underline{b}}) = \prod _{k=0}^{\ell -1}{\delta \bigg (d(\mathbf {\underline{b}}) \le d(\mathbf {\underline{b}} \oplus 2^k)\bigg )} \end{aligned} \end{aligned}$$

for single bit sequence $\textbf{b}$ and its length is $\ell$ ($\ell _{\textrm{N}}$ for natural and $\ell _{\textrm{G}}$ for Gray labeling), where *δ*() is 1 if the argument is true and 0 otherwise. $\oplus 2^k$ flips the *k*-th bit only to obtain a similar single-bit sequence separated by the Hamming distance of 1 from $\textbf{b}$.

**Fig. 2:**
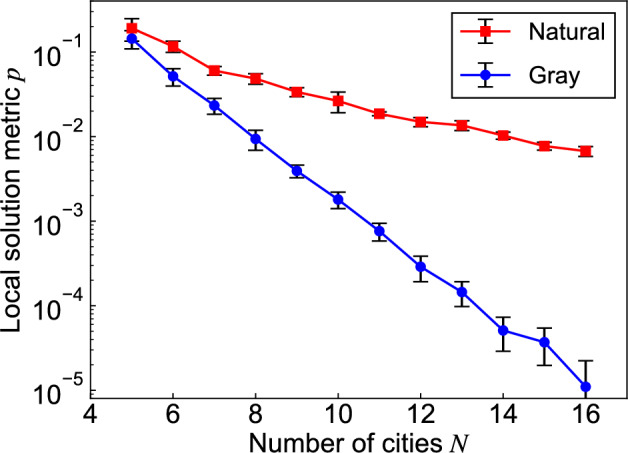
Local solution metric *p* for two labeling methods as a function of the number of cities, *N*. 10 different random configurations of *N* cities were generated and the average values with error bars were plotted. We sampled $2^{\ell }$ cases for $N \le 9$, and $10^5$ random cases for $N \ge 10$ in each configuration.

An example of computing *f* for $N=5$ is provided below. When we treat $\mathbf {\underline{b}}=00110$, the corresponding route $\textbf{r}$ is (2, 1, 3, 4) in natural labeling and (4, 1, 3, 2) in Gray labeling. The set of $\mathbf {\underline{b}} ^\prime =\mathbf {\underline{b}} \oplus 2^k$ is {00111, 00100, 00010, 01110, 10110}, and the set of $\textbf{r}$ is thus {(2, 1, 4, 3), (1, 4, 2, 3), (1, 3, 2, 4), (3, 2, 1, 4), (4, 3, 1, 2)} given by $l_{\textrm{N}}^{-1}(\mathbf {\underline{b}}^{\prime })$ for natural labeling and {(1, 4, 3, 2), (1, 3, 2, 4), (4, 1, 2, 3), (4, 3, 1, 2), (4, 2, 3, 1)} given by $l_{\textrm{G}}^{-1}(\mathbf {\underline{b}}^{\prime })$ for Gray labeling. An arbitrarily similar route $\textbf{r}^{\prime }$ to the reference route $\textbf{r}$ is obtained by swapping a pair of consecutive cities visited. In Gray labeling, any bit sequence from $\textbf{r}^{\prime }$ is described by either $\mathbf {\underline{b}}^{\prime }$, corresponding to the Hamming distance between $\mathbf {\underline{b}}$ and $\mathbf {\underline{b}}^{\prime }$ of exactly 1. This feature is unique to Gray labeling.

Based on *f*, the local solution metric *p* is given by

9 $$\begin{aligned} \begin{aligned} p = \mathbb {E}_{\textbf{b}} \left[ f(\mathbf {\underline{b}}) \right] , \end{aligned} \end{aligned}$$

where $\mathbb {E} \left[ \cdot \right]$ denotes the expectation. Figure 2 shows metric *p* in each labeling for $N = 5$ to 16. For each value of *N*, we generated 10 different random configurations of *N* cities and plotted the average values with error bars. There are too many cases to quantify full ${2^{\ell }}$ cases for $N \ge {10}$; therefore, we sampled a maximum of $10^5$ cases randomly to check whether each bit sequence corresponded to a local minimum. Metric *p* decreases with increasing *N*, where Gray labeling shows a more rapid decrease than natural labeling. This feature is advantageous for better convergence in optimization because it avoids local solutions when exploring solutions through bit flips with an annealing machine. A related challenge in machine learning is the vanishing gradient problem. However, in our approach, we specifically focus on arranging the objective function values to be similar for inputs with similar features, thereby improving the estimation accuracy of the FM. The logic is that by enhancing the FM model’s accuracy, the solutions obtained through annealing will more effectively converge toward the vicinity of the global minimum. We will verify this effect experimentally in the following section.

**Fig. 3:**
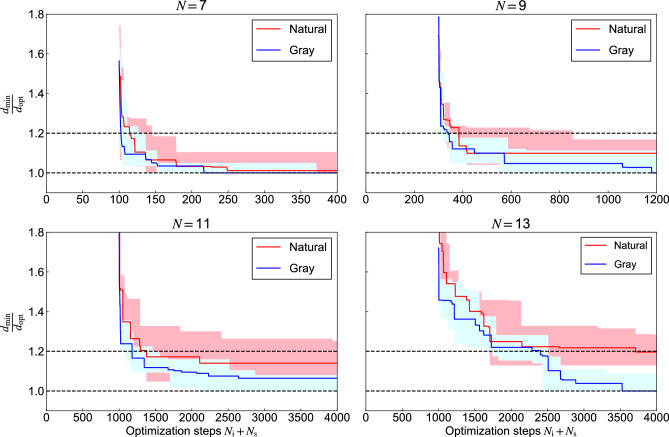
Numerically obtained shortest distance $d_{\textrm{min}}$ until the step normalized by the optimum one in FMA-based TSP for $N =$ 7 to 13. For each value of *N*, 5 random city configurations were generated. The shaded regions represent the range between minimum and maximum values of solutions found across these 5 trials, while the lines show the median values at each step. Simulations were performed with parameters (*N*, $N_\textrm{i}$, $N_\textrm{s}$) = (7, 100, 300), (9, 300, 900), (11, 1000, 3000), (13, 1000, 3000).

## Numerical simulations

This section numerically compares natural labeling and Gray labeling with the FMA in terms of the obtained solution quality and convergence speed. As shown in the preliminaries section, a solution candidate for single-bit sequence $\textbf{b}$ is randomly generated and used in bits $\textbf{x}$ for initial training. After training, an objective function *y* is constructed using the FM. Subsequently, the bit sequence $\textbf{b}$ that minimizes the objective function *y* of Eq. (2) is estimated using an annealing machine, and the route-distance pair is added to the training data. In this study, we used D-Wave quantum annealing machine Advantage System 6.4 (standard QPU) with a default annealing time of 20 µs^37^. The number of data points for the initial training and solution search are denoted by $N_\textrm{i}$ and $N_\textrm{s}$, respectively. These parameters were set to (*N*, $N_\textrm{i}$, $N_\textrm{s}$) = (7, 100, 300), (9, 300, 900), (11, 1000, 3000), (13, 1000, 3000).

**Fig. 4:**
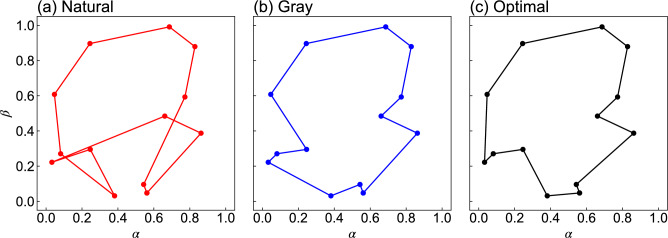
Numerically obtained results of TSP routes in 13 cities: routes obtained by (**a**) natural labeling and (**b**) Gray labeling compared with (**c**) the optimal route.

**Table 3:** The time-to-solution analysis, which shows the number of cities *N*, the number of iterations $N_\textrm{s}$ required to reach the threshold $d_\textrm{min}/d_\textrm{opt} = 1.2$ and the corresponding computational time.

*N*	Natural		Gray	
	$N_\textrm{s}$	Time (min)	$N_\textrm{s}$	Time (min)
7	15	0.35	3	0.06
9	85	1.98	40	1.07
11	376	10.65	179	4.80
13	2713	99.48	1401	63.51

The results of the comparison between the two labeling methods are presented in Fig. 3. Here, $d_\textrm{opt}$ and $d_\textrm{min}$ indicate the globally optimum and minimum distances obtained until the step, respectively. We utilized the Held-Karp algorithm^25^ to find the globally optimum distances based on Eq. (3). For each condition, simulations were conducted with 5 different random city configurations, and the median of the results is shown as solid lines, while the range between maximum and minimum values is indicated by shaded areas. Gray labeling mostly shows a smaller $d_{\textrm{min}}$ or faster convergence than natural labeling in any optimization step for every *N*. For the cases of $N =$ 7, 9, and 11, Gray labeling achieved global optimal solutions in terms of median values, whereas natural labeling reached the global optimum only in the $N = 7$ case. Regarding minimum values, Gray labeling consistently achieved global optimal solutions across all tested cases, while natural labeling succeeded only in the $N = 7$ case.

Figure 4 shows the finally obtained routes by (a) natural and (b) Gray labelings at the final optimization step in one of our trials, and (c) the globally optimal route for $N = 13$. The corresponding distances, *d*, were 4.48, 3.34, and 3.23, respectively. Compared with the optimal route, natural labeling and Gray labeling yielded routes longer by 39% and 3%, respectively. The time-to-solution analysis is presented in Table 3, which shows the number of cities *N*, the number of iterations $N_\textrm{s}$ required to reach the threshold $d_\textrm{min}/d_\textrm{opt} = 1.2$, and the corresponding computational time. Natural labeling requires approximately twice as many iterations as Gray labeling to achieve the same solution quality. The time per iteration is nearly identical for both labeling methods within each city case. The increase in iteration time with larger *N* is primarily attributed to the computational overhead of FM estimation, while QA results are obtained within a few seconds for all cases. The computational efficiency could be further improved by implementing multicore CPU processing for FM estimation.

Overall, Gray labeling is expected to avoid local solutions more frequently than natural labeling, resulting in a better quality-speed balance, as predicted from the local solution metric described in the previous section.

## Conclusion

This study addresses local solution characterization and its avoidance using the bit labeling method in FMA, a QUBO solver combined with machine learning. In particular, we focused on TSP, where FMA can reduce the required number of bits from $N^2$ to $N\textrm{log}N$ for *N*-city TSP. Within the context of the FMA-based TSP, natural and Gray labelings were compared. Natural labeling converts $(N-1)!$ routes to the lexicographical integer and straight-binary label, whereas Gray labeling employs an inversion number and Gray coding to realize the idea of *similar bits for similar routes* using a slightly larger number of bits. The originally introduced metric simply quantified the local solution ratio without performing actual optimization, where Gray labeling showed a rapid reduction in the ratio compared to natural labeling as increasing *N*. Through actual numerical optimization, Gray labeling often shows a better balance of solution quality and convergence speed because of the feature of a lower probability of being captured at local solutions. Our results suggest that both the proposed Gray labeling and metric are useful for QUBO solvers combined with machine learning, such as FMA.

## Data Availability

The details of the numerical simulations are available from S.K. on reasonable request.
